# Drug-dominated dopamine circuits spiral addicts down to a cognitive/behavioral conflict: a neurocomputational theory

**DOI:** 10.1186/1471-2202-13-S1-O8

**Published:** 2012-07-16

**Authors:** Mehdi Keramati, Boris Gutkin

**Affiliations:** 1 Group for Neural Theory, Inserm U960, Ecole Normale Superieure, Paris, France

## 

Long-term addicts find themselves powerless to resist drugs, despite knowing that drug-taking may be a harmful course of action, and an explicit motivation to quit. In controlled experiments, human addicts show a self-described mistake characterized by an inconsistency between drug-seeking response and their reported subjective value. We provide a unified computational theory for this inconsistency by showing how addictive drugs gradually produce a motivational bias toward drug-seeking at low-level habitual decision processes, despite the low abstract cognitive values. This pathology emerges within the hierarchical reinforcement learning (HRL) framework when chronic drug-exposure pharmacologically hijacks the dopaminergic spirals that cascade reinforcement signal down the ventro-dorsal cortico-striatal hierarchy.(1)

**Figure 1:**
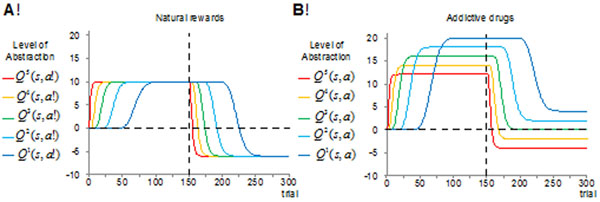
In the first 150 trials, the value of seeking natural rewards at all levels converge to *r*=*10* (a). For the case of drug, however, the direct pharmacological effect of drug (*D*=*2*) results in the value of drug seeking to become much higher than at the detailed levels, than at the abstract levels (b). If both of these actions be followed by punishment of magnitude *-16* (the last 150 trials), then whereas cognitive loops assign a negative value to drug-seeking choice, motor-level loops find drug-seeking desirable (assign a positive value to it).

Here, *r_t_* is the rewarding value of the outcome, be it natural rewards or addictive drugs. These equations show that in order to compute the prediction error signal for updating the value (*Q*) of state-action pairs at the *n*-th level of decision hierarchy, the value of the temporally-advanced state (*s_t_*_+_*_1_*) comes from one higher level of abstraction (*n*+*1*). This captures the role of dopamine-dependent serial connectivity linking the ventral to the dorsal striatum (known as dopamine spirals), which is suggested to integrate information across the segregated cortico-basal ganglia loops, thereby allowing more abstract levels to tune the reinforcement signal used at more detailed levels [1]. The pharmacological effect of addictive drugs on increasing the extracellular concentration of dopamine within the striatum is incorporated into this model by adding a positive term *D* to the prediction error signal. Simulation results (Figure 1) show that drug-induced dopamine-release puts a bias on the transfer of reinforcement signal from one level of abstraction to the next. The accumulation of these biases along the rostro-caudal axis progressively induces a significant discrepancy in the value of drug-seeking behaviors at the top and bottom extremes of the hierarchy, thereby, an inconsistency between cognitive plans and motor-level habits.

Beside this central phenomenon, our model also accounts for several behavioral and neurobiological aspects of addiction, such as the gradual insensitivity of drug-seeking to drug-associated punishments (compulsivity), the delayed development of cue-elicited dopamine efflux in addicts’ dorsal striatum, and the occurrence of blocking effect for drug rewards. It also suggests key testable predictions and beyond that, sets the stage for a view of addiction as a pathology of hierarchical decision making processes.
